# Iodixanol Development of a Laboratory-Scale Technique to Monitor the Persistence of Mycobacterium avium subsp. paratuberculosis in Cheddar Cheese

**DOI:** 10.1100/tsw.2003.109

**Published:** 2003-12-03

**Authors:** J. A. Donaghy, N. L. Totton, M. T. Rowe

**Affiliations:** Agriculture, Food and Environmental Science Division (Food Microbiology Branch), Department of Agriculture and Rural Development for N. Ireland, Belfast, N. Ireland, UK

**Keywords:** Mycobacterium avium subsp, paratuberculosis, Cheddar cheese, dairy, milk, persistence

## Abstract

Mycobacterium avium subsp. paratuberculosis (Map) is a potential human pathogen known to be present in raw milk from infected dairy herds. Current pasteurisation regimes do not totally inactivate Map resulting in the possibility of viable cells being present in pasteurised milk used for Cheddar cheese production. A laboratory-based method, ensuring strict safety precautions, was developed to manufacture 800-g Cheddar blocks, experimentally contaminated (postpasteurisation) with two different strains of Map. The composition of the model Cheddar produced was consistent with commercial product. Syneresis of the cheese curd caused a 1 log10 concentration of Map numbers from milk to cheese for a strain isolated from pasteurised milk. The type strain NCTC 8578 did not show a similar concentration effect, but did however survive the Cheddar manufacturing process. A small percentage (<5%) of the Map load for each strain was recovered in the whey fraction during the process.

## INTRODUCTION

*Mycobacterium avium* subsp. *paratuberculosis* (Map) is the known cause of Johne's disease, a chronic granulomatous enteritis in ruminants and other animals[1,2]. Crohn's disease, an intestinal inflammatory disorder that affects humans, shows many similarities to Johne's disease and Map has been isolated from humans suffering from Crohn's disease[3,4,5,6,7]. However, it is still unknown whether the association of Map and Crohn's disease is coincidental or causal.

Dairy cattle infected with Map are known to secrete the bacterium in their milk and furthermore shed Map through their faeces[8,9]. Therefore, raw milk from an infected herd is likely to be contaminated with Map[10]. Map is more resistant to adverse conditions such as low pH, high temperature, and salt than most other pathogenic bacteria[11]. Several studies investigating the heat resistance of Map have demonstrated the ineffectiveness of normal pasteurisation regimes including high-temperature, short-time (HTST) pasteurisation to totally kill Map present in raw milk[12,13,14,15,16,17,18,19,20]. A more recent study has provided firm evidence of the presence of viable Map in commercially pasteurised cows' milk manufactured for retail sale[21]. Therefore, if Map is present in raw milk and is not effectively inactivated by pasteurisation, there is a possibility that the microorganism may be present in a viable form in other dairy products such as cheese. The UK Government has advocated a precautionary approach and has supported actions to minimise exposure of the public to Map.

Since pasteurisation has been shown to be ineffective in eliminating this potentially human-pathogenic mycobacterium, efforts have concentrated on modifying the time and temperature parameters of pasteurisation to improve its lethality for Map in liquid milk. However, these modifications, whilst appropriate for liquid milk, may affect Cheddar cheese yield and textural properties and therefore it is likely that normal pasteurisation methods or even those with a lower lethality will continue to be used for milk used in Cheddar cheese production. The inactivation of Map in cheese milk will also depend on the environmental conditions of Cheddar manufacture and maturation *viz a viz* cheese cooking temperature, low moisture, low pH, and prolonged low-temperature ripening. In order to assess the effect of these conditions on the persistence of Map, it is necessary to manufacture Cheddar cheese on a laboratory scale to simulate commercial production. Health and safety constraints have prevented routine laboratory-scale production to investigate the fate of pathogenic bacteria during Cheddar cheese manufacture. There is a need, therefore, for the development of a laboratory-scale technique for the controlled production of Cheddar cheese to assess the fate of Map or other pathogens during the manufacturing and ripening stages.

To the authors' knowledge, the only previous work investigating Map survival in cheese was carried out by Sung and Collins[11], who prepared a soft Hispanic-style cheese under laboratory conditions and Spahr and Schafroth[22], who used a pilot-scale process to manufacture model hard (Swiss Emmentaler) and semihard (Swiss Tisliter) cheese. There has been no published research on the survival of Map in Cheddar cheese, the major hard cheese produced worldwide. The method described, based on that developed by Banks and Muir[23], was adapted to ensure rigorous safety precautions were maintained to allow monitoring of Map persistence during the manufacture of Cheddar cheese.

## MATERIALS AND METHODS

### *Mycobacterium avium* subsp. *paratuberculosis* Strains

Two strains of *M. avium* subsp. *paratuberculosis* were tested: a type strain NCTC 8578 (bovine source) and strain 806PSS, which was originally isolated from pasteurised cows' milk (kindly provided by Dr. I. Grant, Food Science Division, Queens University of Belfast, Belfast, N. Ireland).

Each strain was cultured in 100 ml of Middlebrook 7H9 broth medium containing 10% (w/v) Middlebrook OADC (oleic acid-albumin-dextrose-catalase) (Becton Dickinson UK Ltd, Oxford), 0.5% (v/v) Tween 80 (Sigma, UK), and 0.0002% (w/v) mycobactin J (Synbiotics Europe SAS, Lyon, France) for up to 8 weeks. Cell suspensions were homogenised with a Vortex Genie 2 (Scientific Industries Inc., Bohemia, NY) for 3 min to disperse large clumps of Map and decimally diluted in maximum recovery diluent (MRD, Oxoid, Basingstoke, UK) prior to inoculation of the cheese milk.

## Cheddar Cheese Production

All antibiotic-free milk used for cheesemaking was collected and commercially pasteurised on-farm at 72°C for a minimum of 15 sec on the morning of cheesemaking. Milk samples were tested for the presence of Map on the test media below. All Cheddar cheeses were manufactured according to traditional procedures[24] using rennet (Hannilase XL 205, Chr. Hansen, Berkshire, UK) and the mesophilic/thermophilic homofermentative starter culture YY80 (Chr. Hansen). Ten grams of frozen direct vat set starter was dispersed in 90 ml MRD and 0.009% (v/v) used to inoculate the cheese milk. Titratable acidity of milk and whey was used to monitor the manufacturing process[24]. A total of six trials was performed comprising triplicate cheeses for each Map strain.

The cheesemaking apparatus was similar to that used by Banks and Muir[23]. The rectangular stainless steel vat (305 × 200 × 200 mm, capacity 10 l), with a centrally sloping floor, was fitted with a motorised paddle to stir the milk during cheesemaking. The vat was completely enclosed with a stainless steel jacket with a uniform 25-mm space all round, through which water was circulated by an electronically controlled flow heater (Julabo F34, Jencons, Leighton Buzzard, UK). Cheese knives and a cheddaring box similar to those used by Banks and Muir[23] were used for the coagulum cutting and cheddaring processes, respectively. Cheeses were pressed in a Moorlands cheese press (Type S2, Moorlands Cheesemakers, Somerset, UK) at a pressure of 50 kPa for 16 h at 10°C. The pressed curd was removed from the moulds, sampled for microbiological analysis (see below), and vacuum packed (Mini Jumbo, Henkelman Vacuum Systems, Holland) in food-compatible vacuum packages.

Strict safety precautions were taken to prevent any infection of personnel or contamination of the environment. All work was performed in a Containment level 2 laboratory. The cheese vat was located in a Class 1 biological safety cabinet (200 × 70 × 60 cm, Rayair, Mach-Aire, Bolton, UK) and was positioned on two laboratory jacks allowing elevation of the vat during the “whey run” stage of cheese manufacture (Fig. 1). All manipulations of inoculated cheese milk, curd, and whey were performed inside the biological safety cabinet. All cheese utensils were sterilised by autoclaving before use and whey was disinfected in a 5%(v/v) Trigene solution (Medichem International, Kent, UK).

The Cheddar manufacturing process indicating the Map inoculation and sampling points are outlined in Fig. 2. For all cheeses manufactured, 8 l of pasteurised milk was inoculated with 8 ml of an appropriately diluted culture of Map

## Microbiological Analysis

Sterile cheese irons (1 × 10 cm) were used to remove cheese plugs from the pressed cheese blocks 24 h after manufacture. Two representative plugs (5.0 g) from inside the cheese block were transferred to sterile stomacher bags containing 45 ml cheese diluent. The cheese diluent comprised 0.5% (w/v) sodium chloride, 1% (w/v) casitone (Difco, Detroit, MI) and 2.0% (w/v) sodium citrate[20]. The mixture was homogenised at medium speed in a Stomacher peristaltic lab-blender 400 (Seward Medical, London) for 2 min and incubated at 37°C for 1 h submerged in a water bath. The resultant suspension was decimally diluted in maximum recovery diluent (MRD) prior to microbiological analysis.

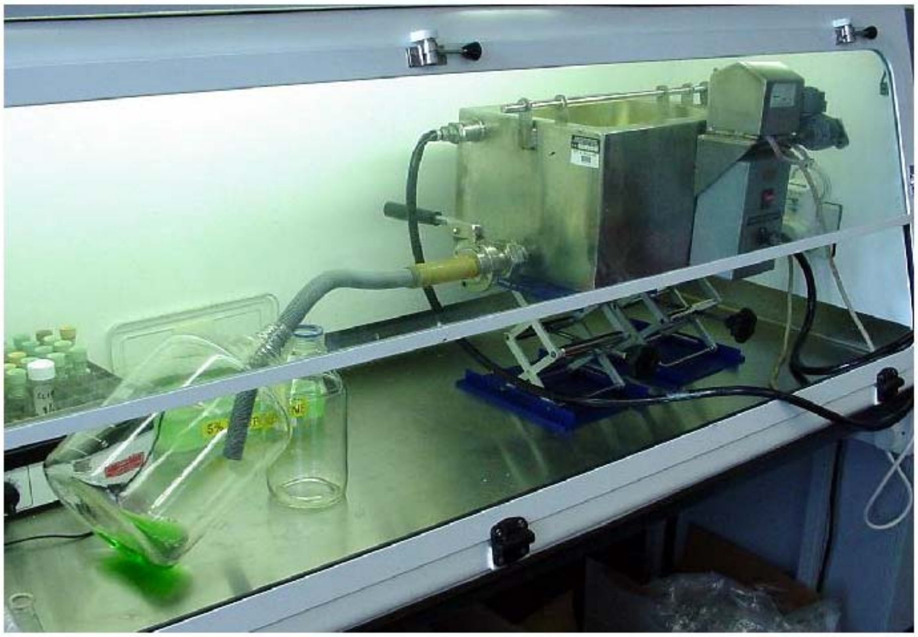

FIGURE 1

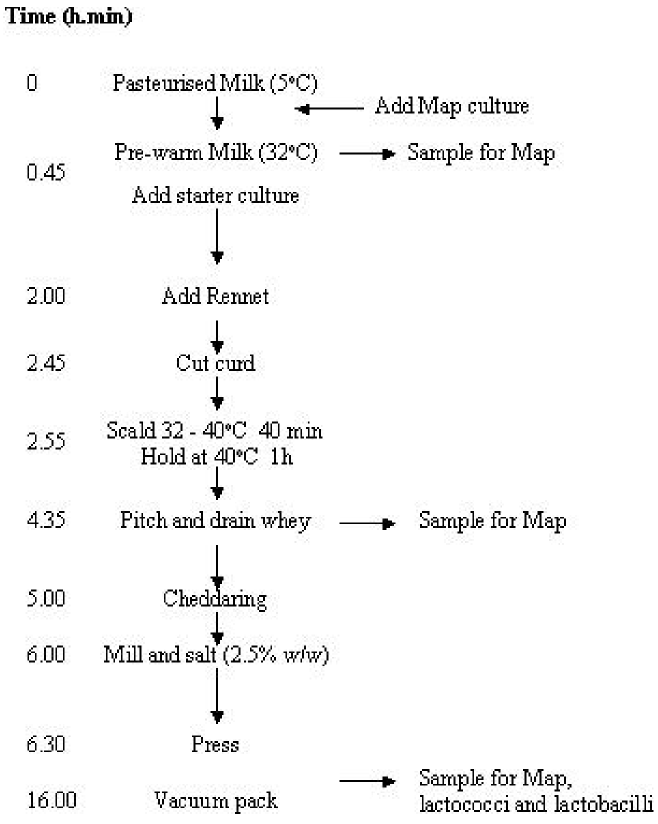

FIGURE 2

Appropriate dilutions of Map culture, inoculated milk, whey, and Cheddar cheese suspensions were inoculated onto Herrold's egg yolk medium (HEYM) (Bacteriological Peptone (Difco,UK), 9 g/l; NaCl, 4.5 g/l; Bacteriological Agar No.1 (Oxoid), 15.3 g/l; beef extract (Sigma, UK), 2.7 g/l; sodium pyruvate (Sigma, UK), 4.1 g/l; glycerol (Sigma, UK), 27 ml/l; malachite green (Sigma, UK), 2% (w/v), 5 ml/l; and sterile egg yolk, 100 ml/l supplemented with an antibiotic cocktail (VAN) containing vancomycin (8.4 µg $ml^{-1}$), amphotericin B (16.8 µg $ml^{-1}$), and nalidixic acid (25 µg $ml^{-1}$) (all Sigma, UK). Agar slopes were incubated at 37ºC for up to 12 weeks and examined periodically for the presence of colonies. The presence of Map was confirmed on representative colonies by acid-fast staining and by the polymerase chain reaction (PCR) for the characteristic insertion element IS900[25]. Starter lactic acid bacteria were enumerated using MRS agar (Oxoid, Basingstoke, UK) and Rogosa agar (Oxoid) was used to monitor changes in lactobacilli numbers. Both media were incubated at 30ºC (5% $CO_{2)}$, for a minimum of 48 h.

## Cheese Compositional Analysis

Standard procedures as described by Kirk and Sawyers[26] were used to analyse cheeses for pH, moisture, salt, and fat (Gerber method).

## RESULTS AND DISCUSSION

A protocol for the preparation of Cheddar cheese, experimentally infected with Map, under controlled microbiological conditions was successfully designed. The method involved the containment of a cheese vat and associated operations within a biological safety cabinet, thereby protecting personnel and the laboratory environment from infection with this Hazard Group 2 bacterium. The method used the technical specification of the cheese-making apparatus designed by Banks and Muir[23]. Uniform Cheddar cheese blocks (766 +/– 20 g) were recovered from 8 l of cheese milk (9.7% yield). The cheese yields recorded in this study were similar to those recovered by Banks and Muir[23], who reported organoleptic properties for the model cheeses, equivalent to commercial cheeses. The composition of the cheese blocks was consistent with good quality Cheddar produced commercially (Table 1). The high lactococcal ($\log_{10}$ 10.3 cfu $g^{-1}$) and lactobacilli ($\log_{10}$ 7.0 cfu $g^{-1}$) counts recorded on day one of manufacture reflect the nature and inoculum of the starter used. The compositional parameters of pH, salt in moisture, moisture in nonfat substance, and fat in dry matter compared favourably with the values for first- and second-grade Cheddar according to the Cheddar classification guide used by Fitzimons et al.[27].

Although time consuming, requiring 6–7 h to manufacture an unpressed cheese block, the method could be repeated on alternate days allowing disinfection and resterilisation of cheesemaking apparatus.

The developed method was used to monitor the persistence of Map during the Cheddar manufacturing process. Although Sung and Collins[11] reported the *in vitro* effects of NaCl and pH (two factors contributing to the inactivation of bacterial pathogens during cheese manufacture and ripening) on the inactivation of Map, there have been no reports on the survival of Map during Cheddar cheese manufacture. Results from our model cheeses indicate the survival of both Map strains during the Cheddar manufacturing process (Table 2). The recovery of Map from cheese milk infected with 3.8 +/– 0.47 $\log_{10}$ cfu $ml^{-1}$ (isolate 806PSS) and 3.6 +/– 0.43 $\log_{10}$ cfu $ml^{-1}$ (NCTC 8578) were 4.8 +/– 0.47 $\log_{10}$ cfu $g^{-1}$ and 3.8 +/– 0.51 $\log_{10}$ cfu $g^{-1}$ from the respective curds. This concentration of Map is expected due to syneresis of the curd during Cheddar manufacture where approximately 90% of the microbial population will be entrapped in the curd.

**TABLE 1**

**Compositional Analysis of Cheddar Cheese Spiked with *M. avium* subsp. *paratuberculosis***

| | Mean Value | Standard Deviation | Cheddar Classification Range (%) | |
|---|---|---|---|---|
| | | | First Grade | Second Grade |
| Lactococci (log $_{10}$ cfu $g^{-1}$) | 10.3 | 0.16 | | |
| Lactobacilli (log $_{10}$ cfu $g^{-1}$) | 7.0 | 0.30 | | |
| Yield (g) | 766 | 20.10 | | |
| Yield (%) | 9.7 | 0.25 | | |
| pH | 5.2 | 0.03 | | |
| Salt (% w/w) | 1.55 | 0.05 | | |
| Moisture (% w/w) | 37.2 | 0.46 | | |
| Fat (% w/w) | 32.1 | 1.10 | | |
| Salt:Moisture (%) | 4.1 | 0.11 | 4.7–5.7 | 4.0–6.0 |
| Moisture:Nonfat substance (%) | 54.2 | 0.78 | 52–54 | 50–56 |
| Fat:Dry matter (%) | 50.5 | 1.50 | 52–56 | 50–57 |

**TABLE 2**
**Survival of *M. avium* subsp. *paratuberculosis* During the Manufacture of Cheddar Cheese**

| Strain/Isolate | . Trial No. | Seed Culture (6–8 weeks) | Map Count $\log_{10}$ cfu $ml^{-1}/g^{-1}$ | | | |
|---|---|---|---|---|---|---|
| | | | Expected Milk Inoculum | Actual Milk Inoculum | Whey | Cheese Curd (24 h) |
| 806PSS | 1 | 7.3 | 4.3 | 4.2 | 2.8 | 5.3 |
| | 2 | 7.3 | 4.3 | 4.2 | 2.6 | 5.0 |
| | 3 | 6.4 | 3.4 | 3.2 | 1.4 | 4.2 |
| NCTC 8578 | 1 | 6.9 | 3.9 | 4.0 | 0.7 | 4.4 |
| | 2 | 6.6 | 3.6 | 3.8 | 1.4 | 3.9 |
| | 3 | 6.4 | 3.4 | 3.0 | 1.0 | 3.1 |

Spahr and Schafroth[22] estimated that 100 cfu of Map per litre of cheese milk would result in a concentration of bacteria to 90 cfu per 100 g of cheese after 24 h. Isolate 806PSS showed an approximate tenfold increase in Map numbers from cheese milk to curd, indicating no inactivation of this strain during Cheddar manufacture. The type strain NCTC 8578 did not show the same proportional increase in Map numbers indicating a possible reduction in this Map isolates' numbers during the manufacturing process. This may be due to the wild type strain, isolated from pasteurised milk, having a greater resistance to the various manufacturing parameters; pH, salt, moisture, cooking temperature, and the antagonistic cheese microflora, than the type strain used. Strain differences have been demonstrated previously by Sung and Collins[11], who investigated the combined *in vitro* effects of pH and salt on the inactivation of Map.

The results provide evidence that both strains survived simulated Cheddar cheese manufacture albeit at high initial inoculum levels. Other studies on the survival of several species of pathogenic bacteria — *Salmonella enterica, Aeromonas hydrophila, Escherichia coli, Campylobacter jejuni,* and *Listeria monocytogenes* — demonstrated the rapid death of these pathogens at various stages during the first 24 h of hard cheese manufacture[28]. The persistence of Map during the manufacture of Swiss Emmentaler, a hard cheese[22] and Queso fresco, a Hispanic-style soft cheese[11] has been reported. Both these cheeses vary considerably in their manufacturing conditions from Cheddar. Unlike Cheddar, the manufacture of Swiss Emmentaler involves the addition of rapidly growing propionic acid bacteria to cheese milk and a comparatively higher curd-cooking temperature than Cheddar. It has been speculated that both factors may contribute to the death rate of mycobacteria during the manufacture and ripening of cheese[22]. Cheddar cheese contaminated with *M. bovis* has also been shown to be infective for up to 220 days[29]. This is the first report on the persistence of Map during the manufacturing stage of Cheddar production. However, this study did not examine the persistence of Map, at these inoculum levels, throughout the subsequent ripening process. Cheddar cheese produced for retail is generally ripened for a minimum of 3–4 months (mild) and for 12 months and beyond for the mature variety. Therefore, further investigation of the persistence of Map, at least up to 3–4 months postmanufacture is required to assess the risk to the cheese consumer. Additionally, lower levels of Map inoculation could be used to better represent low Map numbers which may persist in cheese milk postpasteurisation. This study has also shown the release of up to 4% of the Map load from the cheese milk into the whey fraction during the manufacturing process (Table 2). This may be an important factor when evaluating the use and safety of whey as a cheese processing by-product and wastewater treatment processes.

## CONCLUSION

A laboratory-scale method has been developed for the manufacture of up to 1-kg blocks of Cheddar cheese, experimentally contaminated with Map. The method alleviates the risk of contamination to personnel or the environment from the pathogenic bacterium being investigated. During Cheddar production a number of factors will contribute to the inactivation of Map or other pathogenic bacteria. These factors will act synergistically to produce a combined bacteriocidal effect greater than each factor assessed on an *in vitro* basis. This method has enabled the *in vivo* assessment of this combined effect on the inactivation of Map during Cheddar cheese production. Map has been shown to concentrate and survive in the cheese curd during manufacture with a smaller percentage of the Map load released through the whey fraction. The method developed will permit routine studies on the persistence of Map or other food pathogens during Cheddar cheese manufacture and ripening. Although Map cells in their exponential growth phase were used to contaminate pasteurised milk in this study, the method could be modified to perform studies with heat-treated Map cells. This would authenticate the possible presence of sublethally injured Map in cheese milk postpasteurisation. An *in vivo* assessment of other cheese-making parameters such as starter variation, addition of adjunct lactobacilli, salt levels, pH levels, cooking temperatures, and ripening conditions on Map inactivation could be investigated further with this protocol. Furthermore, the developed protocol could be used to monitor the survival of other pathogens during cheese manufacture.
